# The Molecular Chaperone GRP78/BiP in the Development of Chemoresistance: Mechanism and Possible Treatment

**DOI:** 10.3389/fphar.2013.00010

**Published:** 2013-02-11

**Authors:** Corinna Roller, Danilo Maddalo

**Affiliations:** ^1^Institute of Toxicology and Genetics, Karlsruhe Institute of TechnologyEggenstein-Leopoldshafen, Germany

**Keywords:** cell stress, chaperone, unfolded protein response, drug resistance, therapy

## Abstract

Treatment of several types of cancer such as lung, breast, prostate, and pancreas has shown notable progresses in the past decades. However, after an initial response, tumors eventually became resistant to chemotherapy. This phenomenon, known as chemoresistance, accounts for the death of most cancer patients. Several studies in patients refractory to therapy have revealed the upregulation of the molecular chaperone GRP78/Binding Protein, BiP (BiP) both at the RNA and protein expression level. Furthermore GRP78/BiP relocates to the cell membrane in malignant but not in benign cells. In this communication we review studies on the role and the mechanism of action of GRP78/BiP during development of chemoresistance in cancer cells. In addition we discuss the possible role of GRP78 as a biomarker and as a target in cancer therapy.

## Introduction

One of the features of chemotherapeutic drugs in the control of cell growth is their ability to trigger the stress response. When tumors become refractory to therapy in the course of treatment with chemotherapeutic drugs, proteins involved in stress-regulatory pathways are usually found upregulated. One of these proteins is the 78-kDa glucose regulated protein GRP78 (also known as Binding Protein, BiP). GRP78/BiP is a member of the Hsp70 family of chaperones and localizes mainly in the endoplasmic reticulum (ER; Haas and Wabl, 1983; Hendershot et al., 1994; Hendershot, 2004). The characteristic feature of GRP78 is its dual role in the ER: on one hand it functions as resident chaperone regulating protein folding and preventing aggregation; on the other hand it regulates a pathway known as the unfolded protein response (UPR) through the binding to the ER transmembrane proteins Activating Transcription Factor 6 (ATF6), Inositol REquiring protein 1 (IRE1), and PKR-like Endoplasmic Reticulum Kinase (PERK; Hendershot et al., 1994; Hendershot, 2004). Accumulation of unfolded peptides titrates GRP78 away from these three “stress sensors” inducing their activation. Once activated, the UPR can be divided into two phases: early, pro-survival and late, pro-apoptotic UPR. Intriguingly it is often observed that genes up- or downstream the UPR are upregulated in several type of cancers (Ma and Hendershot, 2004) suggesting that chronic activation of this pathway results in a growth advantage for tumors.

GRP78 is not found exclusively in the ER but it can also localize in the cytoplasm and at the cell membrane. Re-location of GRP78 has been associated to development of drug resistance and cell transformation. In addition it has recently been shown that a splice variant of GRP78/BiP, know as BiPva, lacks the N-terminal ER localization sequence and that this alternative splicing is specific to cancer cells (Ni et al., 2009). Considering the role GRP78 plays both inside and outside the ER in chemoresistance development it is therefore urgent finding compounds inhibiting its pro-survival action.

## GRP78/BiP as Main Regulator of Cell Stress

### GRP78 in the endoplasmic reticulum

**Figure 1:**
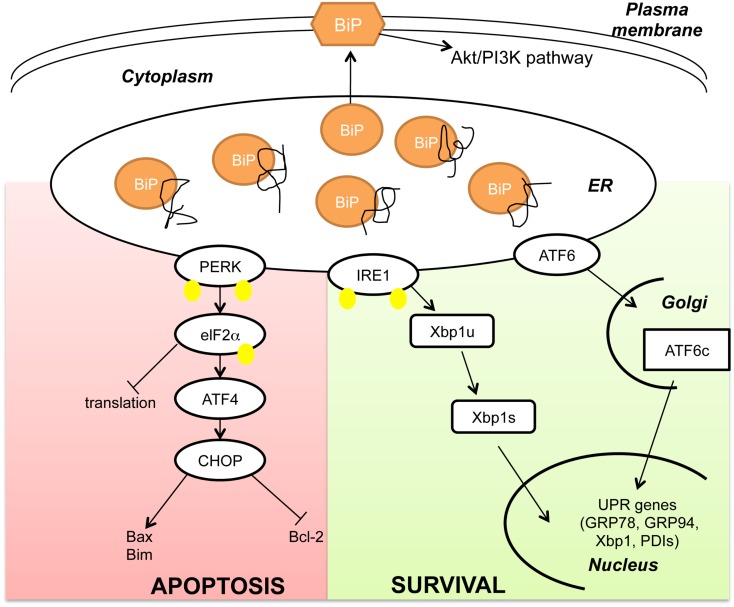
**BiP as master regulator of the Unfolded Protein Response**. Upon accumulation of unfolded peptides in the Endoplasmic Reticulum (ER) the chaperone BiP is titrated away from the stress sensors PKR-like ER kinase (PERK), inositol-requiring protein 1 (IRE1) and activating transcription factor 6 (ATF6). When released by BiP, PERK homodimerizes and autophosphorylates before phosphorylating the alpha subunit of the eukaryotic initiation factor 2 (eIF2α). eIF2α phosphorylation inhibits transcription while activating the transcription factor ATF4 and its target gene C/EBP homologous protein (CHOP). CHOP induces apoptosis by suppressing Bcl-2 and activating Bim and Bax gene expression. Similarly to PERK, the kinase/endonuclease IRE1 homodimerizes and autophosphorylates before splicing Xbp1 mRNA (Xbp1u: Xbp1 unspliced; Xbp1s: Xbp1 spliced). The third branch of the UPR is activated after cleavage of the transcription factor ATF6 in the Golgi (cATF6). Both cATF6 and Xbp1s regulate transcription of pro-survival genes like Glucose Regulated Proteins 78 and 94 (GRP78 and 94) and Protein Disulfide Isomerases (PDIs). In addition to its canonical function in the ER, it has also been shown that BiP can localize to the plasma membrane and regulate the Akt/PI3K pathway. (Yellow dots indicate phosphorylation. For simplicity PERK and IRE1 are shown as monomers).

The ER is a cellular organelle where proteins are folded and/or modified prior to their export either to the cytoplasm or the cell membrane or the extracellular matrix. Stressful conditions, such as exposure of cells to chemicals, lack of nutrients or hypoxia induce protein aggregation, and misfolding in the ER. GRP78/BiP, as ER-resident chaperone, has the dual function of regulating protein folding on the one hand and activating the UPR in acute stress conditions on the other hand. In normal conditions, GRP78 is bound to and inhibits three distinct transmembrane proteins that are also known as stress sensors: the kinase PERK, the kinase/endonuclease IRE1, and the transcription factor ATF6 (Wang et al., 2009). Upon accumulation of unfolded proteins within the ER, GRP78 is titrated away from these stress sensors leading to their activation. The transmembrane kinase/endonuclease IRE1 gets activated after a step of homodimerization and autophosphorylation before splicing the mRNA of the transcription factor X-box Binding Protein 1 (Xbp1; Cox et al., 1993). The spliced form of Xbp1 induces transcription of a specific subset of genes coding for proteins that play a role in ER-mediated peptide folding. Similarly the transcription factor ATF6 in its GRP78 unbound from translocates into the Golgi where it is cleaved and activated (Wang et al., 2000). Activated ATF6 can induce transcription of the molecular chaperones GRP78 and GRP94 as well as of Xbp1, indicating a cross-talk between these two arms of the UPR (Yoshida et al., 2001). The transmembrane kinase PERK homodimerizes, undergoes autophosphorylation and inhibits the alpha subunit of the eukaryotic initiation factor 2 (eIF2α). As a result *de novo* protein synthesis is blocked preventing novel polypeptides from accumulating in the ER lumen (Koumenis et al., 2002). However, prolonged eIF2α phosphorylation leads to the activation of the transcription factor ATF4, a member of the cAMP-responsive element-binding protein (CREB) family of basic zipper-containing proteins (Scheuner et al., 2001). ATF4 induces the transcription factor CHOP/GADD134 (Fawcett et al., 1999) that in turn induces the pro-apoptotic protein Bax (Yamaguchi and Wang, 2004) and Bim (Puthalakath et al., 2007) and inhibits the anti-apoptotic protein Bcl-2 (McCullough et al., 2001). Based on the time of activation, the UPR has opposite effects on cell fate: while at the early stage it induces cell survival and increases refolding activity within the ER by activating ATF6 and IRE1 branches, at later time points results in cell death induction by activating the PERK-eIF2-ATF4 axis (Figure 1).

### GRP78 outside the endoplasmic reticulum: The right balance for chemoresistance

Immunohistochemical as well as genome wide analysis on patient material have shown that GRP78 is overexpressed in several tumors refractory to therapy like glioma (Pyrko et al., 2007), leukemia (Uckun et al., 2011), prostate (Daneshmand et al., 2007), and breast cancer (Scriven et al., 2009). Increased level of GRP78 seems to be the key feature for cancer cells that chronically upregulate the UPR without however inducing apoptosis. The observation that GRP78 is localized at the cell membrane in malignant but not benign cells indicates that the extra-ER function of this protein is crucial for its pro-survival action. Even if it is not clear yet how GRP78 induces chemoresistance, two mechanisms of action could be hypothesized: one possibility is the synergistic effect of both the UPR pro-survival branch and the receptor-mediated activation of the Akt/PI3K pathway. Alternatively the pro-apoptotic action of the UPR could be compensated by the activation of the Akt/PI3K pathway, resulting in cell survival.

Since the UPR is frequently upregulated in refractory tumors and GRP78 plays a crucial role in its activation, it is expected that cancers with higher GRP78 levels will respond less to chemotherapy. Based on this observation GRP78 is therefore a good candidate biomarker to predict patient response to therapy on one hand and a good target to overcame acquired resistance on the other hand.

## GRP78/BiP as Biomarker in Chemoresistant Tumors

One of the characteristic features of cancer cells is their ability to develop resistance to chemotherapeutic agents. This property translates into an initial response of the patients to therapy. However within a period that can vary from some months to years cancer grows back and is unresponsive to the initial therapy. GRP78/BiP has been found overexpressed both at the gene and at the protein level at this stage. For example GRP78 is found commonly overexpressed in breast cancer lesions refractory to therapy (Gazit et al., 1999; Fernandez et al., 2000). Treatment of human breast cancer cells MDA-MB-435 with anti-angiogenic factor Combretastatin A4P showed increased expression of GRP78/BiP in the surviving cells, indicating that higher GRP78 levels correlate to higher resistance (Dong et al., 2005). In addition GRP78 increased expression has been observed in a panel of MCF-7 human breast cancer cell line refractory to several treatments compared to the parental line (Wosikowski et al., 1997). Intriguingly it has been shown that while high GRP78 levels are predictive of a shorter time to recurrence in stage II and III breast cancer patients treated with the topoisomerase inhibitor doxorubicin (Lee et al., 2006; Scriven et al., 2009), in patients treated sequentially with doxorubicin and taxanes GRP78 is positively associated with better outcome (Lee et al., 2011). These observations indicate that GRP78 could be a specific marker to predict doxorubicin resistance in breast cancer.

In addition it has been shown that GRP78 induces chemoresistance development in brain endothelial cells, favoring therefore tumor vascularization and metastatic spread (Virrey et al., 2008) and that GRP78 inhibition re-sensitize acute lymphoblastic leukemia cells refractory to vincristine (Uckun et al., 2011). GRP78 has also been found to contribute to castration resistant prostate cancer (CRPC) development (Tan et al., 2011). Immunohistochemical analysis has shown that GRP78 levels are overexpressed in CRPC (Pootrakul et al., 2006) and its expression levels positively correlate with poor survival recurrence (Daneshmand et al., 2007). Even if no clear mechanism of action has been described, it has been showed that knockout of GRP78 blocks Akt-PI3K pathway, resulting in tumor growth inhibition (Fu et al., 2008).

## Targeting GRP78/BiP in Refractory Cancers

Since GRP78/BiP is overexpressed in malignant cells resistant to therapy, it is a valid target to overcame chemoresistance. An additional advantage in targeting GRP78/BiP is given by the observation that it translocates on the plasma membrane of malignant but not benign cells, offering the possibility of cancer specific drug delivery with functionalized nanoparticles. Table 1 shows a summary of the compounds described in the following section.

**Table 1:** **List of GRP78 inhibitors**.

	Reference
**ANTIBODIES**	
C38	de Ridder et al. (2012)
C107	
anti-CDT	Misra and Pizzo (2010)
SAM-6	Brändlein et al. (2007)
**NATURAL COMPOUNDS**	
EGCG	Ermakova et al. (2006)
Subtilase toxin AB5	Paton et al. (2006)
Versipelostatin	Matsuo et al. (2009)
Prunustatin A	Umeda et al. (2005)
**PEPTIDES**	
Bag-1 peptide	Maddalo et al. (2012)
M4 peptide	Cunningham et al. (2005), Gupta et al. (2006)
Pep42	Yoneda et al. (2008)
WIFPWIQL	Arap et al. (2004)
WDLAWMFRLPVG	
GIRLRG	Passarella et al. (2010)

The drugs designed to target GRP78/BiP can be divided into three major categories: (1) Antibodies, (2) Natural compounds, (3) Peptides.

### Antibodies

Specific antibodies targeting GRP78 either in the ER or on the cell surface have successfully demonstrated reduction in tumor growth and proliferation. The mouse monoclonal antibody C38 recognizes the C-terminal domain of the murine GRP78 exposed on the cell membrane inhibiting PI3K/Akt proliferative pathway in melanoma cells (de Ridder et al., 2012). A similar mechanism of action has been demonstrated for the antibody C107 in a melanoma mouse model (de Ridder et al., 2012). The anti-CDT GRP78 antibody binds the cell expressed GRP78 in human prostate cancer cells significantly reducing tumor growth (Misra and Pizzo, 2010). Treatment of several prostate cancer cell lines with this antibody showed increased p53 protein levels as well as induction of the pro-apoptotic proteins Bad, Bax, and Bak. Another antibody, SAM-6 (Brändlein et al., 2007), binds to an O-glycosylated form of GRP78 expressed on the cell surface of cancer cells but its anti-growth effect has not been tested yet (Rauschert et al., 2008).

### Natural compounds

The green tea extract (−)-epigallocatechingallate (EGCG) binds the ATPase domain of GRP78 inhibiting its catalytic activity. In addition it prevents GRP78 oligomerization, crucial for the correct function of the chaperone. Administration of EGCG has shown re-gain of sensitivity to the topoisomerase inhibitor etoposide in breast cancer cells (Ermakova et al., 2006). Moreover it has been shown that several cytotoxins inhibit GRP78 either by direct cleavage, like Subtilase AB5 derived from *E. coli* (Paton et al., 2006), or reducing its expression levels like Versipelostatin (derived from *Streptomyces* *versipellis*; Matsuo et al., 2009) or Prunustatin A, a compound isolated from *Streptomyces violaceusniger* (Umeda et al., 2005).

### Peptides

Peptides binding GRP78 are employed either for direct inhibition of GRP78 activity or to coat nanoparticles to deliver cytotoxic drugs.

One of the direct inhibitors of GRP78 is the Bag-1 peptide, derived from a novel interaction site on the co-chaperone Bag-1 that binds GRP78 C-terminal domain (Maddalo et al., 2012). Bag-1 peptide ectopic expression in several malignant but not benign prostate cancer cell lines as well as in prostate cancer xenograft models reduces tumor growth by inhibiting GRP78 refolding activity and inducing CHOP-mediated apoptosis. Similarly the M4 peptide was derived from the Mda-7 tumor suppressor protein after the observation that Mda-7 intratumoral gene transfer was able to reduce growth of several cancers (Cunningham et al., 2005; Gupta et al., 2006).

The evidence that GRP78 is selectively expressed on the surface of several cancer types gives the possibility of exploiting molecules that can bind GRP78 for selective drug delivery. A good example is given by the cyclic peptide Pep42 (CTVALPGGYVRVC) that can bind selectively to surface GRP78 and can function as cell penetrating peptide (Yoneda et al., 2008). Also the peptides WIFPWIQL and WDLAWMFRLPVG are able to bind GRP78 on the cell surface and have been employed successfully in *in vivo* models of prostate and breast cancer for the delivery of a cell death-inducing peptide (Arap et al., 2004). GRP78-binding peptides can be used also in conjugation to other carriers like liposomes or nanoparticles. In fact the peptide WIFPWIQL has been used conjugated to liposomes for doxorubicin delivery to cancer endothelial cells and in an *in vivo* colon carcinoma mouse model (Katanasaka et al., 2010). Similarly the peptide GIRLRG was employed for coating nanoparticles containing placitaxel in irradiated breast carcinomas and showed increased cell death compared to known chemotherapy approaches (Passarella et al., 2010).

## Conclusion and Perspectives

The role of GRP78 in development of chemoresistance is just emerging. The increasing availability of genome wide and array data shows that GRP78 is often overexpressed in several types of cancers refractory to conventional therapy. GRP78 therefore could not only be a good biomarker to predict response to therapy but also an appealing target for a more selective chemotherapy.

## Conflict of Interest Statement

The authors declare that the research was conducted in the absence of any commercial or financial relationships that could be construed as a potential conflict of interest.
